# Assessing Military Mental Health during the Pandemic: A Five Country Collaboration

**DOI:** 10.1007/s11920-024-01522-3

**Published:** 2024-10-12

**Authors:** Jennifer E. C. Lee, Clare Bennett, Neanne Bennett, Fethi Bouak, Irina Goldenberg, Kate Harrison, Heather McCuaig Edge, Amy Millikan Bell, Phillip J. Quartana, Maj Amos Simms, Amy B. Adler

**Affiliations:** 1 Director General Military Personnel Research and Analysis, Department of National Defence, Ottawa, ON Canada; 2 Defence Health Directorate, New Zealand Defence Force, Wellington, New Zealand; 3 Joint Health Command, Australian Defence Force, Canberra, NSW Australia; 4 Defence Research Development Canada – Toronto Research Centre, Toronto, ON Canada; 5 Defence Statistics (Health), Ministry of Defence, Bristol, UK; 6 Defense Centers for Public Health – Aberdeen, Aberdeen Proving Ground, Maryland, USA; 7 Center for Military Psychiatry and Neuroscience, Walter Reed Army Institute of Research, Silver Spring, Maryland, USA; 8 Academic Department of Military Mental Health, King’s College London, London, UK

**Keywords:** Pandemic, Armed forces, Military personnel, Mental health, Workplace well-being, Employee surveys, Leadership

## Abstract

**Purpose of Review:**

Members of a technical panel representing Australia, Canada, New Zealand, the UK, and the US collaborated to develop surveys designed to provide military leaders with information to guide decisions early in the COVID-19 pandemic. The goal of this paper is to provide an overview of this collaboration and a review of findings from the resulting body of work.

**Recent Findings:**

While surveys pointed to relatively favorable mental health and perceptions of leadership among military personnel early in the pandemic, these observations did not reflect the experiences of personnel deployed in COVID-19 response operations, nor were these observations reflective of later stages of the pandemic.

**Summary:**

Establishing and leveraging networks that enable the rapid development of employee surveys and sharing of results can serve as a pathway for empowering military leaders in times of crisis. Organizational support and leadership decisions are especially critical for maintaining well-being among personnel during crises.

## Introduction

There is a long history of military engagement to support relief efforts during disease outbreaks, given the ability of militaries to leverage their training and equipment to rapidly mobilize resources and establish capabilities [1–5]. Thus, it was not surprising that countries engaged their militaries to support response efforts over the course of the COVID-19 pandemic. Across the Five Eyes countries (i.e., Australia, New Zealand, Canada, UK, US), examples of such efforts included the provision of logistic support to civilian agencies, support to hospitals and long-term care facilities, border protection, as well as support to quarantine, testing, contact tracing, and vaccination programs [6–10].

Despite the breadth of experience that could be leveraged to support the response to COVID-19 among military personnel, the unprecedented nature of the crisis pushed many into uncharted territory. To date, over 6.9 million people have died due to COVID-19 worldwide, and there has been upwards of 700 million confirmed cases [11]. Early in the pandemic, there was intense uncertainty, shifting guidelines, and fear related to the threat of an unpredictable and deadly infection. Some personnel had to adapt quickly to working from home under less-than-ideal conditions, while those involved in COVID-19 response were at increased risk of exposure, resulting in concerns about putting loved ones at risk. As personnel adjusted to this new reality, they faced anxiety in their daily lives as the broader impacts of the pandemic became apparent [12]. Concerns regarding the cumulative impact of these challenges prompted military leaders to examine the mental health and resilience of their organizations.

The way organizations respond to crises contributes to enduring perceptions amongst personnel about the degree to which the organization cares for them. Managing crises effectively and compassionately can help organizations build trust and organizational commitment among personnel [13]. Leaders can play a key role in effectively managing workplace well-being during crises, by optimizing decision-making, providing timely and transparent communication, and demonstrating empathy [14]. For this to be possible, however, they must be equipped with situational awareness of challenges faced by their personnel. Nowhere is this commitment to crisis management more evident than in the military context, where the psychological contract between the organization and serving personnel is paramount. This psychological contract is an implicit agreement that the organization will support its service members (e.g., train, equip, lead, and address health-related needs). In exchange, individuals agree to serve their country even if that means putting themselves at risk [15]. Thus, the military expects leaders to address the concerns of their personnel.

In order to inform leaders’ decision-making, organizations often turn to employee surveys. Besides assessing workplace climate and indicators of change, employee surveys provide personnel with an outlet to identify their concerns [16, 17]. Such surveys have routinely been used by military organizations across the Five Eyes countries [18–22]. Accordingly, each country was familiar with the potential value of administering a survey to guide military leaders during the COVID-19 crisis. Given the unique nature of the COVID-19 pandemic, there was no established model for how to best approach such surveys. As a result, international counterparts were able to rely on one another for rapid lessons learned.

Early in the pandemic, discussions about employee surveys to support organizational responses to COVID-19 emerged among members of an ongoing technical panel within The Technical Cooperation Program (TTCP), a collaboration involving the Five Eyes countries. This technical panel was centered on exploring the role of leadership in promoting behavioural health through sustained support, modeling, and communication. Consequently, the group was well situated to extend the concept of behavioural health leadership to the management of the COVID-19 pandemic in the military community. Through sharing materials and resources, surveys were rapidly developed and administered in each nation to identify challenges faced by military personnel and potential targets for intervention by leaders. The purpose of the present paper is to provide an overview of this collaboration, along with a review of findings that emerged from the resulting body of work.

## Method

Technical panel members developed the surveys in parallel, making it possible to include overlapping content and carry out cross-national comparisons. For instance, the US and Canada assessed probable anxiety and depression with similar tools (more details presented below), and the UK and Canada used the same item to assess change in mental health since the start of the pandemic. As well, similar “checklist” approaches were used in Canada, New Zealand, and the US to identify key pandemic-related challenges faced by personnel. In alignment with panel members’ common interest in the role of leadership in promoting behavioural health, surveys from each country also included questions on perceptions of leadership during the pandemic. Notwithstanding similarities across the surveys, however, there were important differences in some of the content, as well as the eventual timing, scope, frequency, analysis, and reporting of the surveys to suit each country’s unique needs. In particular, reflecting the diverse nature and timing of pandemic-related military operations across countries, surveys in some countries (e.g., Australia) focused entirely on personnel involved in such missions, while those in others (e.g., Canada) explored levels of readiness for potential operations.

To shed light onto the experiences of military personnel during the COVID-19 pandemic across the countries, a review of the resulting body of work was a carried out. The review included any publication in which results of the surveys were presented (i.e., internal briefings or reports, conference presentations, or journal articles), and emphasized content in which the surveys overlapped. Table 1 provides a summary of the characteristics of surveys from each country, along with a list of publications that were available for the review. A synthesis of key observations is presented below. Even though surveys overlapped in content, comparisons between countries must be interpreted with caution, considering differences in the timing, scope, frequency, analysis, and reporting of the surveys across the countries.

**Table 1:** Summary of survey characteristics and relevant publications

**Survey** **Title**	**Initial** **Administration**	**Sample** **Characteristics**	**Themes** **Assessed**	**Additional Administrations**	**Relevant** **Publications**
**Australia**					
OP-COVID19 Deployment Experiences Survey	November 2020	A total of 1,852 Australian Defence Force (ADF) members completed this survey (78% men, 21% women), including Army (55%), Navy (31%), and Air Force (14%) personnel, representing a response rate of 18%.	– COVID-19 deployment – Operational concerns – Morale – Health and well-being – Pandemic concerns		Chesney C, Bennett N. Operation COVID-19 Assist – Results of the Deployment Experiences Survey. Presentation at the AMMA Conference; 2022 Apr; Melbourne, Australia.
**Canada**					
COVID-19 Defence Team Survey	April–May 2020	Over 27,000 individuals completed the survey, including 13,668 Canadian Armed Forces (CAF) Regular Force and 5,985 Reserve Force members (of which, 5,052 were Primary Reserve members). Most Regular Force and Primary Reserve members were men (82% and 80%, respectively). Regular Force members were slightly older, with the majority of them falling between the ages of 25 and 44 years (65%) and the majority of Primary Reserve members aged 34 years or less (60%). Almost half (49%) of Regular Force members and just over two-fifths (61%) of Primary Reserve members were junior non-commissioned members.	– COVID-19 deployment – COVID-19 exposure – Health and well-being – Pandemic concerns – Household impacts – Stress management – Social support – Leadership support – Communication – Access to healthcare – Support programs – Personal protection	Additional surveys administered in: – March–May 2021 (N = 1,743 Regular Force members; N = 1,004 Primary Reserve members) – September–November 2022 (N = 1,928 Regular Force members; N = 843 Primary Reserve members; results not reported here)	Blais AR, Comeau C, Daugherty C, Goldenberg I, Frank C, Guérin E., et al. Covid-19 Defence Team Survey. Ottawa, Ontario, Canada: Defence Research and Development Canada; 2020. DRDC-RDDC-2020-D104. Goldenberg I, Lee JEC. Spring 2021 Your-Say Survey: COVID-19 results. Briefing to the Department of National Defence/Canadian Armed Forces Total Health and Wellness Strategy Sub-Committee; 2021 Dec. Goldenberg I, Lee JEC, Blais AR, Comeau C, Frank C, Guérin E, et al. COVID-19 Defence Team Survey: Top-line findings. Ottawa, Ontario, Canada: Defence Research and Development Canada; 2020. DRDC-RDDC-2020-R084. Lee JEC, Goldenberg I, Blais AR, Comeau C, Daugherty C, Frank C, et al. Trials and tribulations among members of Canada’s Defence Team early in the pandemic: Key insights from the COVID-19 Defence Team Survey. Health Promotion and Chronic Disease Prevention in Canada: Research, Policy and Practice. 2022 Mar;42(3):104 [25]. Sudom K, Guérin E, Lee JEC. Gender-related differences in mental health of Canadian Armed Forces members during the COVID-19 pandemic. Journal of Military, Veteran and Family Health. 2021 Sep;7(S1):46–57 [24].
**New Zealand**					
NZDF Covid-19 Wellbeing Check	May 2020	A total of 3,006 members of the New Zealand Defence Force (NZDF) Community completed the survey, of which 1,705 comprised Regular Force members (representing a 19% response rate, of whom 59% were Army). Ranks across the Regular Force were represented, with 62% non-commissioned officers and 38% commissioned officers.	– COVID-19 deployment – Operational concerns – Health and well-being – Pandemic concerns – Household impacts – Stress management – Leadership support – Access to healthcare – Support programs – Communication	Additional surveys administered in: – August 2020 (N = 2,611) – March–April 2021 (N = 1,999)	Heather-Smith K. Things that have been bothering me: (Regular Force top 5) [Unpublished Figure]; 2022. New Zealand Defence Force. NZDF COVID-19 Wellbeing Check (C-19WC) T1 preliminary report. NZDF; 2020. New Zealand Defence Force. NZDF COVID-19 Wellbeing Check 3 - Preliminary results: Regular Force and civilian. NZDF; 2021.
**United Kingdom**					
COVID-19 Survey	July–August 2020	The survey received just over 7,600 valid responses from a convenience sample of members of the Defence Community (military and civilian personnel). Over half were military personnel, including Regular Force members (43%) and Reservists (9%), of whom 44% were officers.	– Morale – Health and well-being – Pandemic concerns – Household impacts – Work outcomes – Stress management – Social support – Leadership support – Communication – Support programs		Flecknoe K, Harvey J, Bodman S, Smith P, Harrison K. COVID-19 Survey Appendix 1: MOD breakdown by employment type / gender / grade / TLB. Ministry of Defence briefing; 2020 Nov; United Kingdom. Harvey J, Bodman S, Flecknoe K, Smith P, Harrison K. COVID-19 Survey: Insights and next steps. Ministry of Defence briefing; 2020 Oct; United Kingdom.
**United States**					
Behavioral Health Assessment Team (BHAT) - COVID-19 Assessment	May–June 2020	A total of 21,911 US Army Soldiers completed the survey, resulting in an aggregate response rate of 28% across three major commands throughout the Asia–Pacific, Europe, and Northwest US Region. Most participants were male (85%), between the ages of 17 and 29 years (59%), and 50% were junior enlisted personnel. The remaining participants who specified their rank comprised of either senior enlisted personnel (33%) or Warrant Officers/Officers (14%).	– COVID-19 exposure – Health and well-being – Pandemic concerns – Household impacts – Leadership support – Communication – Access to healthcare – Personal protection – Growth	Surveys administered to same respondents in: – April 2021 – July–August 2021 – November–December 2021 – February–March 2022 (results not reported here)	Adler AB, Gutierrez IA, Gomez SAQ, Beymer MR, Jackson Santo T, Thomas JL, et al. US soldiers and the role of leadership: COVID-19, mental health, and adherence to public health guidelines. BMC Public Health. 2022 Dec;22(1):1–9 [27]. Quartana PJ, Beymer MR, Gomez SAQ, Adler AB, Jackson Santo T, Thomas JL, et al. COVID-19 concerns, information needs, and adverse mental health outcomes among U.S. Soldiers. Military Medicine. 2024 Mar/Apr;189(3–4): e878-e887 [26]. Quartana PJ, Millikan Bell A, Adler AB, Jackson Santo T, Gomez SAQ, Beymer MR, et al. Behavioral Health Advisory Team – COVID-19 Survey phase 1 findings. Walter Reed Army Institute of Research; 2020 Nov. Technical Report No. S.0079120–20 [23].

## Results

### Mental Health and Well-being

As shown in Table 1, all surveys assessed health and well-being, although the specific measures and analyses varied. In both Canada and the US, it was found that 12% of Primary Reserve and 15% of Regular Force CAF members, and 16% of US Army soldiers met the criteria for probable anxiety, while 12% of Primary Reserve and 14% of Regular Force CAF members, and 17% of US Army soldiers met the criteria for probable depression [23]. Probable anxiety and depression were assessed using the two-item Generalized Anxiety Disorder (GAD-2) and two-item Patient Health Questionnaire (PHQ-2) screening tools in both countries, respectively, although different criteria were applied. Cutoff scores of 3 on each tool were used to indicate probable anxiety and depression in Canada, whereas participants were deemed to meet the criteria for probable anxiety or depression in the US only if they obtained scores of 3 or more on the tools and reported at least some symptom-related functional impairment on a related item from the full PHQ. Among US Army soldiers, these rates were in line with pre-COVID-19 comparison samples using similar or identical indicators, and lower than those observed in combat deployed environments, such as during Operation Enduring Freedom or Operation Iraqi Freedom [23].

Regarding perceived changes in mental health, results were reported for all Regular Force members in Canada, and separately for male and female Regular Force members in the UK. Nevertheless, findings in Canada were similar to those of the UK. Specifically, 36% of CAF Regular Force members reported worse mental health since the start of the pandemic, while 40% female and 32% male Regular members of the UK Armed Forces reported such changes. Conversely, 16% of CAF Regular Force members reported better mental health since the start of the pandemic, which was comparable to the 22% of female and 16% of male Regular members of the UK Armed Forces.

Results in the NZDF indicated relatively favourable mental health among military personnel, with 86% reporting they were “feeling healthy, happy and satisfied in life” or “mostly feeling happy and healthy”. Only roughly 1% of both Regular and Reserve Force members indicated they had been “really struggling” and 12% of Regular Force and 10% of Reserve Force members indicated they felt they had been “struggling a bit” over the last week. Furthermore, only 2% of Regular Force and less than 1% of Reserve Force members reported having new concerns about their mental health.

Collectively, results did not point to sizeable mental health concerns early in the pandemic; however, other findings emphasized factors associated with higher risk. Specifically, among personnel deployed in response to the pandemic, 22% of ADF personnel deployed on Op COVID-19 Assist reported at least moderate psychological distress based on a score of 11 or more on the 6-item Kessler Psychological Distress Scale – a higher rate than was seen for Op Bushfire Assist and most overseas operations in 2020, suggesting that involvement in COVID-19 operations may have had a unique impact on mental health.

In addition, women reported poorer mental health compared to men across most of these countries. Among CAF Regular Forces members, women were more likely than men to meet the criteria for probable anxiety and probable depression. They were also more likely to indicate that their mental health and stress levels had gotten worse since the start of the pandemic [24]. Similarly, US Army female soldiers had greater odds of meeting the criteria for probable anxiety (odds ratio [OR] of 1.59, 95% confidence interval [CI]: 1.41, 1.80) and probable depression (OR of 1.25, 95% CI: 1.10, 1.41) compared to male soldiers [23], and female Regular UK Armed Forces respondents were more likely to report worse mental health (40% versus 32% for male Regular Force respondents).

### Pandemic-Related Challenges

**Fig. 1:**
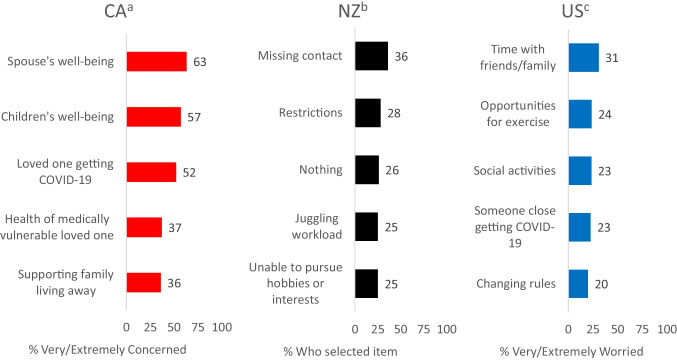
Summary of the top five pandemic-related challenges reported by military personnel in Canada, New Zealand, and the US. ^a^Question was “Please rate the extent to which you are concerned about the following in the context of the COVID-19 pandemic [Not at all, Somewhat, Very, Extremely, N/A]”. Results are for the Regular Force. ^b^Question was “What has been contributing to how you have been feeling? [tick all that apply]”. Results are for the Regular Force. ^c^Question was “Please rate the extent to which you are worried or concerned about the following in the context of COVID-19 [Not at all, Slightly, Moderately, Very, Extremely]”. Results are for US Army soldiers

While surveys in each country assessed pandemic-related challenges (i.e., work, family, health), use of a similar checklist in Canada, New Zealand, and the US enabled comparisons of the relative ranking of these challenges across these countries. Results are summarized in Fig. 1 and described below.

Among CAF Regular Force members, spouse’s/partner’s well-being, child(ren)’s well-being, a loved one getting COVID-19, the health of a medically vulnerable loved one, and supporting family living away emerged as the most prominent challenges [25]. In the NZDF, missing contact with friends and loved ones, restrictions being placed on what people can do, juggling workload and other responsibilities, and not being able to pursue hobbies or interests were items that Regular Force personnel indicated contributed most to how they had been feeling over the last week (although a comparable proportion of personnel indicated nothing had been bothering them). Finally, US Army soldiers most frequently reported being concerned or worried about time with friends/family, opportunities for exercise, social activities, someone close getting COVID-19, and changing rules, regulations, and guidance related to COVID-19 [23]. These concerns were associated with greater odds of a positive mental health diagnosis, and tended to be greater among non-White soldiers [26]. Thus, issues pertaining to the well-being of loved ones, social connection, and pandemic regulations emerged as common themes in terms of pandemic-related challenges faced by military personnel.

Regarding demographic differences, further analyses indicated that the number of reported pandemic-related concerns did not significantly differ by gender among US Army soldiers, with male and female soldiers endorsing an average total number of 23.0 and 24.6 COVID-19 stressors, fears, and concerns, respectively [23]. Among CAF personnel, it was similarly found that Regular Force men and women did not differ in their number of reported pandemic-related concerns. However, this was not the case among Primary Reserve members, where higher numbers were observed among women compared to men [25]. Furthermore, while Regular Force men and women reported a similar number of pandemic-related concerns, they nevertheless differed in the extent to which they reported specific types of challenges, with Regular Force women more likely to report issues related to children and supporting family members (e.g., balancing work and childcare, supporting family members living remotely, school closures, unavailability of childcare) and men more likely to report concerns related to their spouse’s job security.

### Experiences During COVID-19 Operations

For the initial surveys, only the ADF focused on personnel having been deployed in support of the COVID-19 response. However, questions were added in a NZDF follow-up survey, administered in March–April 2021, to assess the experiences of personnel who had been deployed in Op PROTECT.

ADF personnel deployed in Op COVID-19 Assist were asked to describe what they found to be the most stressful about the operation. Participants most commonly cited disorganisation, which was described as a lack of information and role clarity, role changes, and the general “unknown”. Job stress, along with a lack of work and boredom, were also cited as stressful aspects of the deployment, as were general leadership issues. Among NZDF personnel who were deployed in Op PROTECT, key deployment-related stressors included being away from family, fatigue, maintaining fitness, missing out on training/career development, and boredom. Thus, there was some, but not much overlap with the experiences of ADF personnel.

In both the ADF and NZDF, however, it was found that the experiences of personnel during COVID-19 operations also depended on their operational role. For instance, what NZDF personnel reported as the best thing about their work in Op PROTECT varied according to the nature of their role, with those involved in security pickets reporting little satisfaction with their work and those involved in isolation or quarantine facility management/assistance reporting “doing something important” as the best thing about their work. In the ADF, Op COVID-19 Assist roles involving isolation compliance and COVID-19 testing were associated with the highest levels of stress.

### Experiences with Leadership

Most surveys assessed perceptions of leadership, albeit to varying extents. As noted above, issues with leadership were commonly cited by ADF personnel upon being asked what they found most stressful about their deployment in Op COVID-19 Assist.

Outside of operational contexts, however, perceptions of leadership were generally favourable. Most of the surveys included questions examining the level of support that personnel felt leadership was providing them in the COVID-19 context. In most cases, personnel believed their leadership was supportive and effectively managing the pandemic. In Canada, for example, 60% of Regular Force and 57% of Primary Reserve personnel reported receiving significant support from their supervisor, and supervisors ranked among the top three sources of support for personnel, emphasizing the important role that leadership can play in a crisis [25]. In the UK, 77% of Regular Force personnel felt the level of communication they received from their Ministry of Defence about COVID-19 was right. Among members of the overall UK defence community (i.e., military and civilian personnel combined), 82% agreed that their line manager was supportive of work-life balance issues and 75% agreed that they kept them apprised of the latest information. Results were similar across service types of the defence community, although Regular Force members reported lower agreement that their line manager was supportive of work-life balance issues.

Similarly, in the NZDF, 82% reported satisfaction with the support and communication they received from their immediate manager, 74% were satisfied with their access to support services, and 74% were satisfied with communication about their job/role.

Surveys in both Canada and the US examined the extent to which personnel believed their leaders emphasized self care, worked to maintain social connections, and acknowledged the inherent stress and uncertainty of the pandemic. In both countries, most personnel either agreed or strongly agreed with these statements. Specifically, 87% of CAF Regular Force personnel agreed that their supervisor emphasized self-care and acknowledged the stress and uncertainly of the pandemic, while 83% agreed that their supervisor kept them socially connected. Perceptions were similar, although slightly better among Primary Reserve personnel [25]. In the US, more than half of Army soldiers agreed or strongly agreed with these statements (social connection: 59%; acknowledgement of stress/uncertainty: 58%; and emphasis on self-care: 56%) [23].

In the US, further analyses were conducted to examine the association of specific COVID-19 leadership behaviours with behavioural health indicators. Results indicated that more supportive leadership was associated with less likelihood of meeting the criteria for probable anxiety or depression, and greater likelihood of adhering to preventive health guidelines, such as wearing a mask, frequently washing hands for 20 seconds with soap and water, using hand sanitizer, and self-monitoring for symptoms. Specifically, even after adjusting for general leadership, US Army soldiers who reported their immediate supervisor as engaging in high levels of COVID-19 leadership behaviours had roughly half the odds of meeting the criteria for probable anxiety or depression, and over twice the odds of adhering to preventive health guidelines, compared to those who reported their immediate supervisor engaged in low levels of COVID-19 leadership behaviours [27].

### Evolution of Experiences Throughout the Pandemic

To address how the experiences of personnel might have evolved over the course of the pandemic, many of the countries administered employee surveys at additional timepoints. Despite the generally favourable picture of mental health that emerged among personnel outside of the operational context early in the pandemic, results of surveys conducted at later stages of the pandemic suggested that such levels were not sustained over time, emphasizing the importance of continually monitoring workplace well-being as a crisis unfolds.

For US Army soldiers, top COVID-19 concerns continued to involve spending time with friends, social activities, and changing rules, regulations, and guidance related to COVID-19, and there was little evidence these had decreased over a period of 6–9 months [26].

After about one year since the start of the pandemic, roughly half of CAF personnel (i.e., 56% Regular Force and 47% Primary Reserve personnel) indicated that their mental health had gotten worse since September 2020. Similarly, in the NZDF, the percentage of Regular Force personnel who reported that they were really struggling rose steadily from 1% in May 2020 to 2% in August 2020 and to 3% in March–April 2021, as did the percentage who reported struggling a bit, which rose from 12% in May 2020 to 17% in August 2020 and to 22% in March–April 2021.

Regarding pandemic-related challenges, the top five concerns reported by CAF personnel continued to center around the health and well-being of loved ones (i.e., spouse’s/partner’s well-being, child(ren)’s well-being, health of a medically vulnerable loved one). However, these top challenges now included maintaining social ties and limited opportunities for exercise. By comparison, changes in pandemic-related challenges in the NZDF were much more pronounced, with different types of items reported as challenging and “nothing” no longer emerging among the items that personnel most endorsed. Specifically, the top pandemic-related challenges reported by NZDF Regular Force personnel in March–April 2021 still included juggling workload and other responsibilities (reported by 26% of personnel); however, they now also included job satisfaction (reported by 34% of personnel), sleep (reported by 31% of personnel), concern for the well-being of others (reported by 20% of personnel), and finances (reported by 20% of personnel). Thus, at later stages of the pandemic, top concerns seemed to become more varied and cumulative, spanning impacts on behavioural health (i.e., exercise and sleep) and finances.

## Discussion

The unprecedented nature of the COVID-19 pandemic placed significant pressures on organizations worldwide. While military organizations offered job security, compensation and benefits, and health care to personnel when many were facing significant uncertainty regarding their employment and/or financial well-being, their role in supporting COVID-19 relief efforts also meant putting personnel on the frontlines of the crisis. Rapidly developing and launching employee surveys provided military leaders with information that could inform their decisions on how to best support personnel and address their needs in the workplace to sustain their operational readiness.

Patterns in the data across countries point to some key areas to address in future pandemics. First, mental health may be strained in military populations as time goes on. While military populations appeared relatively resilient to the stressors associated with the pandemic early on, observations made at later points showed they are not immune. These impacts may have been compounded by a lack of social connection and inability to engage in certain coping methods (e.g., exercise) due to physical distancing measures, emphasizing the need for programs that can overcome these gaps to minimize strain. These results show the importance of assessing mental health and well-being at multiple timepoints to fully appreciate how the impacts of enduring stressors may evolve over time, and which resources can be leveraged to bolster resilience and strengthen protective factors, such as leadership and social support, when the usual means for doing so are constrained.

Second, several findings suggested that women are at greater risk (or more aware of the impact of strain on their well-being) than men. Indeed, it is believed that pre-existing gender inequalities placed women at a greater risk of experiencing mental health problems during COVID-19, and that gender inequalities concerning family and work have widened [28, 29]. Notwithstanding the importance of these observations, results of systematic reviews have suggested that mental health outcomes were worse by only a small amount for women compared to men among members of the general population, and only for certain conditions (i.e., anxiety and depression) [30, 31]. Moreover, differences in symptoms of depression between men and women have been reduced when using measures that assess externalizing symptoms (i.e., substance use, anger) in addition to traditional symptoms (i.e., sadness, crying) [32]. Thus, it could be important to consider anger or alcohol misuse to better understand the longer-term implications of the pandemic for men in future military employee surveys.

Finally, deploying in support of the community is important to ensure service members feel a sense of purpose [33], but results from the survey of ADF personnel deployed on Op COVID-19 Assist emphasized that it can also place additional strain on the personnel involved. Attention should be placed on understanding the unique challenges faced by personnel who were involved in COVID-19 relief efforts, along with their impact on mental health, to facilitate the development of more targeted interventions and support programs. For instance, issues around moral injury have been found to be common among personnel deployed in support of relief operations, especially among those involved in providing medical support [34].

Recognizing that the success of employee surveys is largely dependent on the development of follow-up or action plans, results were reported to stakeholders and packaged to help military leaders understand how findings might inform decisions. In Canada, results were shared with senior Commanders in a series of internal briefings, and with members of the Defence Team on the Department’s internal website in a series of infographics and a report, which provided a set of tips based on results [35]. In New Zealand, results led to (a) enhanced communications about available support services to the Defence Community, (b) the provision of additional wellness resources for personnel being deployed on Op PROTECT and their families, (c) the development of resources for leaders on managing risks and monitoring workplace well-being, and (d) the development of new resources to address the needs of personnel (e.g., respite care, resilience webinars, provisions for children’s education). In the US, results informed the development of fact sheets and guides to provide military leaders with tips for messaging and decisions regarding the provision of resources related to COVID-19. These were shared widely by military leaders.

## Conclusion

Ultimately, international collaboration through the technical panel proved to be significantly advantageous for supporting organizational responses to COVID-19. Leveraging the panel enabled its members to quickly develop the surveys by drawing on each other's materials. As well, developing the surveys in parallel allowed panel members to include overlapping content, providing opportunities for cross-national comparisons. In turn, this resulted in a collective body of work that could be reviewed to better understand the impacts of the pandemic on military personnel across a broader range of contexts.

To optimize the impact of employee surveys and enhance preparedness in the event of future crises, it is important to sustain international working groups that facilitate rapid collaboration. Even when militaries are different in terms of their size, combat focus, and culture, there are many common elements that benefit from this kind of cooperation. For example, it might also be useful to have repositories of existing validated tools that may be shared for future surveys. This collaboration confirmed that, when implemented and actioned effectively, employee surveys can be a valuable tool to help military leaders manage workplace well-being within their organization in times of crisis [16]. In preparation for the next pandemic, it is important to bear in mind that approaching employee surveys though an international lens is a force multiplier.

## References

[1] Adler AB, Kim PY, Thomas SJ, Sipos ML. Quarantine and the US military response to the Ebola crisis: Soldier health and attitudes. Public Health. 2018;155:95–8. 29331771 10.1016/j.puhe.2017.11.020

[2] Byerly CR. The US military and the influenza pandemic of 1918–1919. Public Health Rep. 2010;125(3_suppl):81–91. PMC2862337 20568570

[3] Kalkman JP. Military crisis responses to COVID-19. J Contingencies Crisis Manag. 2021;29(1):99–103. 10.1111/1468-5973.12328 PMC7537208 40477147

[4] Sipos ML, Kim PY, Thomas SJ, Adler AB. US service member deployment in response to the Ebola crisis: The psychological perspective. Mil Med. 2018;183(3–4):e171–8. 29514338 10.1093/milmed/usx042

[5] Wilén N. The military in the time of COVID-19. PRism. 2021;9(2):20–33.

[6] Australian Government Defence. Operation COVID-19 Assist [Internet]. Australian Government Defence; 2023 [cited 2023 Oct 11]. Available from: https://www.defence.gov.au/operations/covid19-assist.

[7] Government of Canada. Operation LASER [Internet]. Government of Canada; 2023 Jan 9 [cited 2023 Oct 11]. Available from: https://www.canada.ca/en/department-national-defence/services/operations/military-operations/current-operations/laser.html.

[8] Headquarters New Zealand Defence Force. CDF Operational Directive 06/2020 NZDF support to the Government response – 2019 novel coronavirus [Internet]. Headquarters New Zealand Defence Force; 2020 Feb 04 [cited 2023 Oct 11]. Available from: http://nzdf.mil.nz/assets/Uploads/DocumentLibrary/Documents-concerning-NZDF-support-to-All-of-Govt-COVID-19-response-1.pdf.

[9] Ministry of Defence. COVID Support Force: MOD’s contribution to the coronavirus response [Internet]. Ministry of Defence; 2020 Oct 30 [cited 2023 Oct 11]. Available from: https://www.gov.uk/guidance/covid-support-force-the-mods-continued-contribution-to-the-coronavirus-response.

[10] United States Northern Command. Active-duty support to COVID-19 response [Internet]. United States Northern Command [cited 2023 Oct 11]. Available from: https://www.northcom.mil/COVID19/.

[11] World Health Organization. WHO coronavirus 19 dashboard [Internet]. World Health Organization; 2023 Oct 04 [cited 2023 Oct 11]. Available from: https://covid19.who.int/?adgroupsurvey=%7badgroupsurvey%7d&gclid=EAIaIQobChMI7tnPjK_ugAMVP2pvBB2hGwXnEAAYASABEgIaOvD_BwE.

[12] Taylor S. The psychology of pandemics. Annu Rev Clin Psychol. 2022;9(18):581–609. 10.1146/annurev-clinpsy-072720-020131 34780260

[13] VandePol B, Gist R, Braverman M, Labardee L. Strategic specialty partnerships: Enabling the EAP for evidence informed best practices in workplace crisis response. J Work Behav Health. 2006;21(3–4):119–31.

[14] D’Auria G, De Smet A. Leadership in a crisis: Responding to the coronavirus outbreak and future challenges. Psychology. 2020;22(2):273–87.

[15] Adler AB, Castro CA. An occupational mental health model for the military. Mil Behav Health. 2013;1(1):41–5.

[16] Derickson R, Yanchus NJ, Bashore D, Osatuke K. Collecting and reporting employee feedback for large organizations: Tips from the Department of Veterans Affairs. The Psychologist-Manager Journal. 2019;22(2):74.

[17] Schuster C, Weitzman L, Sass Mikkelsen K, Meyer-Sahling J, Bersch K, Fukuyama F, Paskov P, Rogger D, Mistree D, Kay K. Responding to COVID-19 through surveys of public servants. Public Adm Rev. 2020;80(5):792–6. 32836447 10.1111/puar.13246 PMC7283646

[18] Goyne A. Measuring unit effectiveness: What do commanders want to know and why. Australian Army Psychology Research and Technology Group. 2004. Available from: https://citeseerx.ist.psu.edu/document?repid=rep1&type=pdf&doi=78bbdaf73b11c7f79c8530d47ecb6d137b56f96a.

[19] Ivey GW, Blanc JR, Michaud K, Dobreva-Martinova T. A measure and model of psychological health and safety in the workplace that reflects Canada’s national standard. Canadian Journal of Administrative Sciences/Revue canadienne des sciences de l’administration. 2018;35(4):509–22.

[20] New Zealand Defence Force. Protecting and improving mental health and wellbeing in the workplace [Internet]. New Zealand Defence Force [cited 2023 Oct 11]. Available from: https://www.healthandsafety.govt.nz/assets/Documents/NZDF_case_study_-_mental_health-v2.pdf.

[21] Elliott-Mabey N, Davison H. UK armed forces continuous attitude survey: A short history and description of a key strategic information tool. BMJ Mil Health. 2019;165(2):133–5. 10.1136/jramc-2018-001041 30341168

[22] McDonald DP. Using the Defense Organizational Climate Survey (DEOCS) to assess command climate over time. Defence Equal Opportunity Management Institute (DEOMI) Directorate of Research Development and Strategic Initiatives; 2019. Tech Report No. 19-02. Available from: https://apps.dtic.mil/sti/pdfs/AD1083928.pdf.

[23] Quartana PJ, Millikan Bell A, Adler AB, Jackson Santo T, Gomez SAQ, Beymer MR, et al. Behavioral Health Advisory Team – COVID-19 Survey phase 1 findings. Walter Reed Army Institute of Research; 2020 Nov. Technical Report No. S.0079120–20. Available from: https://ph.health.mil/PHC%20Resource%20Library/cv19-bhat-techreport-phase1.pdf. This work presents results that are featured in this paper.

[24] Sudom K, Guérin E, Lee JEC. Gender-related differences in mental health of Canadian Armed Forces members during the COVID-19 pandemic. Journal of Military, Veteran and Family Health. 2021;7(S1):46–57. This work presents results that are featured in this paper.

[25] Lee JEC, Goldenberg I, Blais AR, Comeau C, Daugherty C, Frank C, et al. Trials and tribulations among members of Canada’s Defence Team early in the pandemic: Key insights from the COVID-19 Defence Team Survey. Health Promotion and Chronic Disease Prevention in Canada: Research, Policy and Practice. 2022;42(3):104. This work presents results that are featured in this paper. 10.24095/hpcdp.42.3.04 PMC9022953 35262312

[26] Quartana PJ, Beymer MR, Gomez SAQ, Adler AB, Jackson Santo T, Thomas JL, et al. COVID-19 concerns, information needs, and adverse mental health outcomes among U.S. Soldiers. Military Medicine. 2024;189(3–4): e878-e887. This work presents results that are featured in this paper. 10.1093/milmed/usad350 37715687

[27] Adler AB, Gutierrez IA, Gomez SAQ, Beymer MR, Jackson Santo T, Thomas JL, et al. US soldiers and the role of leadership: COVID-19, mental health, and adherence to public health guidelines. BMC Public Health. 2022;22(1):1–9. This work presents results that are featured in this paper. 10.1186/s12889-022-13345-z PMC9092038 35546398

[28] Wenham C, Smith J, Davies SE, Feng H, Grépin KA, Harman S, Herten-Crabb A, Morgan R. Women are most affected by pandemics—Lessons from past outbreaks. Nature. 2020;583(7815):194–8. 32641809 10.1038/d41586-020-02006-z

[29] Yavorsky JE, Qian Y, Sargent AC. The gendered pandemic: The implications of COVID-19 for work and family. In Working in America 2022 (pp. 305–317). Routledge. 10.1111/soc4.12881 PMC8250288 34230836

[30] Cenat JM, Farahi SM, Dalexis RD, Darius WP, Bekarkhanechi FM, Poisson H, Broussard C, Ukwu G, Auguste E, Nguyen DD, Sehabi G. The global evolution of mental health problems during the COVID-19 pandemic: A systematic review and meta-analysis of longitudinal studies. J Affect Disord. 2022;15(315):70–95. 10.1016/j.jad.2022.07.011 PMC9278995 35842064

[31] Dal Santo T, Sun Y, Wu Y, He C, Wang Y, Jiang X, Li K, Bonardi O, Krishnan A, Boruff JT, Rice DB. Systematic review of mental health symptom changes by sex or gender in early-COVID-19 compared to pre-pandemic. Sci Rep. 2022;12(1):11417. 35794116 10.1038/s41598-022-14746-1 PMC9258011

[32] Martin LA, Neighbors HW, Griffith DM. The experience of symptoms of depression in men vs women: Analysis of the National Comorbidity Survey Replication. JAMA Psychiat. 2013;70(10):1100–6. 10.1001/jamapsychiatry.2013.1985 23986338

[33] Trachik B, Oakey-Frost N, Ganulin ML, Adler AB, Dretsch MN, Cabrera OA, Tucker RP. Military suicide prevention: The importance of leadership behaviors as an upstream suicide prevention target. Suicide Life Threat Behav. 2021;51(2):316–24. 33876487 10.1111/sltb.12707

[34] Ein N, Plouffe RA, Liu JJ, Gervasio J, Baker C, Carleton RN, Bartels SA, Lee JEC, Nazarov A, Richardson JD. Physical and psychological challenges faced by military, medical and public safety personnel relief workers supporting natural disaster operations: A systematic review. Curr Psychol. 2023;28:1–6.

[35] Government of Canada. Covid-19 Defence Team Survey findings [Internet]. Government of Canada; 2022 Nov 21 [cited Oct 11]. Available from: https://www.canada.ca/en/department-national-defence/campaigns/covid-19/mental-health/covid-19-defence-team-survey-findings.html.

